# No evidence for association between pupil size and fluid intelligence among either children or adults

**DOI:** 10.3758/s13423-025-02644-2

**Published:** 2025-02-26

**Authors:** Patricia Lorente, Veera Ruuskanen, Sebastiaan Mathôt, Antonio Crespo, Jonas Radl

**Affiliations:** 1 Department of Social Sciences, Universidad Carlos III de Madrid, C. Madrid, 135, 28903 Getafe, Madrid, Spain; 2 Department of Experimental Psychology, University of Groningen, Groningen, The Netherlands; 3 Department of Basic Psychology II, Faculty of Psychology , Universidad Nacional de Educación a Distancia (UNED), C/ Juan del Rosal 10, Ciudad Universitaria, Madrid, 28040 Spain

**Keywords:** Cognitive abilities, Fluid intelligence, Pupil baseline, Pupillometry, Replication

## Abstract

Recent studies have investigated resting-state, or baseline, pupil size as a general measure of cognitive abilities, based on the earlier finding that larger pupils might be predictive of higher general intelligence or working memory capacity. However, evidence for such relationships has been mixed, and all previous studies thus far have focused on adult samples. The present study adds to this debate by examining the correlation between fluid intelligence and baseline pupil size in a sample of both children (10 years old) and adults (their parents). Importantly, our sample is representative in terms of socioeconomic background, which was not the case in previous studies, thus addressing concerns about sample selection and variability. We did not find evidence for a relationship of fluid intelligence with baseline pupil size or with pupil-size variability for either children or adults. Therefore, our results do not replicate the relationship between cognitive abilities and baseline pupil size as reported in previous research.

**Supplementary Information:**

The online version contains supplementary material available at 10.3758/s13423-025-02644-2.

## Introduction

Measurement of pupil size, or pupillometry, is a valuable scientific tool. The conventional approach is to present subjects with tasks or stimuli and to record their change in pupil size relative to a baseline period, with the assumption that the extent to which the pupil dilates reflects arousal or mental effort (for a review, see Mathôt, 2018). However, a series of recent studies has focused on resting-state, or baseline, pupil size instead. The research question driving these studies is: Does a person’s resting pupil size predict that person’s cognitive abilities, specifically fluid intelligence and working memory capacity?

The hypothesis that the resting-state pupil size is correlated with cognitive abilities is linked to the fact pupil size reflects activity in the locus coeruleus (LC)-noradrenergic (NA) system. The LC is a subcortical hub of noradrenergic neurons that provide the sole bulk of norepinephrine (NE) to the cortex, cerebellum and hippocampus (Aston-Jones & Cohen, 2005). The proposed theory suggests that variations in connectivity between the LC and networks responsible for goal-driven behaviour are linked to variations in cognitive capabilities. According to this hypothesis, improved connectivity results in increased resting pupil size, enhanced attention regulation, and heightened fluid intelligence (Tsukahara & Engle, 2021a; Tsukahara et al., 2021).

The first evidence ever collected for a relationship between cognitive abilities and resting pupil size was provided by Heitz et al. (2008), who found a positive correlation between baseline pupil size and working memory capacity (WMC). This was an incidental finding in a study that was actually about the role of incentives on WMC. Similarly, Van der Meer et al. (2010) found, again incidentally, a positive relationship between baseline pupil size pre-task and fluid intelligence in a study designed to test differences in pupil size during tasks between high and low IQ individuals. Following these incidental findings and given that the original studies were not designed to test this particular relationship between baseline pupil size and cognitive abilities, Tsukahara et al. (2016), from the same lab as the original Heitz study, designed the first study to purposefully study this phenomenon. The study was set to rule out the effect of possible confounders such as mental effort or familiarity with the lab, and they tested the relationship between both WMC and fluid intelligence with baseline pupil size. The authors found that both abilities were positively correlated with baseline pupil size, but found that fluid intelligence had the strongest relationship, suggesting that the relationship between baseline pupil size and WMC may be mediated by fluid intelligence (Tsukahara et al., 2016). Additional evidence for a relationship between resting pupil size and cognitive abilities was provided by Aminihajibashi et al. (2019). This study explored the relationship between WMC and baseline pupil size variability, based on the hypothesis that a higher variability in tonic LC-NE firing might be important for behavioural flexibility, arousal regulation, and ultimately performance in the memory tests. Indeed, it found a significant positive relationship between pupil-size variability and WMC (Aminihajibashi et al., 2019).

Despite this initial series of positive results, attempts to replicate these findings in other labs have not been as successful (e.g., see Unsworth & Robinson, 2017; Unsworth et al., 2019). A meta-analysis by Unsworth et al. (2021) found a near zero correlation between WMC and baseline pupil size, with the only significant moderator of the relationship being the location of the study, such that positive findings tended to come from the same lab. In response to this replication failure, Tsukahara and Engle (2021b) provided a series of conditions they found to be missing in the studies included in the meta-analysis and that, in their view, had to be met in order for their effect to be replicated. One of their two main arguments states that previous studies have been conducted in settings that were too bright. Lighting conditions have an impact on both the average and the between-subject variation of pupil sizes. Specifically, in dark conditions, pupils are wider and there is the most variation between individuals, which is necessary to be able to study the between-subject relationship between baseline pupil size and cognitive abilities. They posit as a reference the values of the Tsukahara et al. (2016) study of 5.92 mm average pupil size and 1.09 mm of standard deviation for adult samples. Secondly, as mentioned above, they argue that the relationship between fluid intelligence is more robust than the one between pupil and WMC, which the meta-analysis focuses on. Indeed, using a sample of eight studies, Unsworth et al. (2021) present tentative results indicating a significant positive relationship between baseline pupil size and fluid intelligence. Tsukahara and Engle (2021b) also mention a few additional factors that could contribute to not finding an effect, such as the sample size of the study or using only a single task to measure WMC. Finally, they argue that samples should be more heterogeneous, which should also provide more variability in terms of cognitive abilities, and thus not consist exclusively of college students.

Particularly regarding the relationship between fluid intelligence and the resting pupil size, Coors et al. (2022) tried to assess several cognitive skills, and although their studies did not corroborate a relationship of baseline pupil size with fluid intelligence, they did find a positive relationship with processing speed. They argue, however, that since the performance of the speed processing test depends, among others, on ocular movements on a screen, the higher baseline pupil size might indicate a greater engagement or preparation for the following task, leading to a better performance. In other words, they cannot rule out the confounding effect of task engagement and effort in both their baseline and processing speed measures. Following attempts to test the positive correlation between fluid intelligence and pupil size have since failed to replicate these findings (Robison et al., 2022; Robison & Brewer, 2022; Robison & Campbell, 2023; Ruuskanen et al., 2024).

Of the aforementioned studies, three used samples of undergraduate students (Robison & Brewer, 2022; Robison & Campbell, 2023; Ruuskanen et al., 2024) with an average age of 19 years old. Robison et al. (2022) used a sample of student aviators instead, with an average age of 23.66 years, and Coors et al. (2022) studied this effect in a varied population of adults that ranged in age from 30 to 61 + years. In other words, most studies present homogeneous populations of tertiary education students, leaving Tsukahara and Engle’s concern about the homogeneity of the replication study samples not adequately addressed. Moreover, whether the relationship between pupil size and fluid intelligence replicates or not in children remains untested.

If we could objectively measure fluid intelligence by measuring the resting pupil size, this would provide a valuable tool for research. For instance, test motivation and effort conflate our current measures of cognitive abilities (Duckworth et al., 2011), so resting state pupil size could be used to proxy IQ. Given the theoretical and practical relevance of being able to measure between-subject variance in fluid intelligence through the pupil size, this paper aimed to contribute to the literature by trying to replicate the effects provided by Tsukahara et al. (2016) and Aminihajibashi et al. (2019), and, indirectly, to test their theory (Tsukahara & Engle, 2021a). To this end, this study analysed the relationship between fluid intelligence and baseline pupil size and its variability in a sample of children (around 10 years of age) as well as their parents. To our knowledge, this is the first study on the correlation between pupil size and fluid intelligence in a sample of healthy children, in which a representative distribution of socioeconomic background is ensured.

Hence, the research question this paper addresses is whether we can replicate the effect of Tsukahara and Engle both in children and in adults. Stated otherwise: *Is fluid intelligence positively associated with baseline pupil size and its variability in children and their parents?*

The hypotheses derived from these questions that the study tested are:

- **H1a.** Fluid intelligence is not positively associated with average baseline pupil size in children.
- **H1b.** Fluid intelligence is not positively associated with the variability of the baseline pupil size in children.
- **H2a.** Fluid intelligence is not positively associated with average baseline pupil size in adults.
- **H2b.** Fluid intelligence is not positively associated with the variability of baseline pupil size in adults.

## Methods

The data presented here were collected as part of a large-scale study on the relationship between socioeconomic status and effort.^1^ The data from this large-scale study are stored in a local repository and are not available due to contractual agreements with the university. The processed data used for this paper can be shared upon request.

### Participants

One hundred dyads of parents and their children attending the fifth grade of primary school were invited to participate in the original large-scale study. Because of technical issues, only data from 96 children and 95 parents were preserved. Of the children, 50 of the 96 were female and they were all in the fifth grade of primary school (average age = 10.36 years, *SD* = 0.6 years). Of the parents, 62 of the 95 were female (average age = 42 years, *SD* = 4.84 years). The selection of participants was outsourced to a specialized recruiting company, which recruited participants according to quota for parental employment status, parental education, and child gender. These quotas were adjusted so as to obtain a representative sample of parents by socio-economic status according to official statistics. Moreover, the goal was to have a balanced sample with regard to the gender of the children. After collecting the data, the distribution regarding employment status was: 77 employed, 9 inactive, and 9 unemployed adult participants. In terms of highest level of educational attainment, there were 51 adult participants with higher education, 23 adult participants with technical vocational education, and 21 adult participants with secondary education recruited. Regarding their ethnicity, 14 out of the 96 parents declared not having been born in Spain. The present study complies with ethical standards of the Declaration of Helsinki and the protocol was approved by the ethics committee from the funding and local institution.

### Procedure

Upon arrival, the participating children, accompanied by their parents, were shown the laboratory where the experiment took place. While the researcher showed the child around, an assistant collected the informed consent for both participants from the parent. Child and parent were then separated. The adult performed the experiment first, so that they would become acquainted with the tasks that their children would perform later on. The experimental design of the original study, from which this paper obtains its data, foresaw the performance of a series of real-effort tasks with the simultaneous collection of pupil data. The participants were reminded of this general design and asked about their vision problems or needs and were seated in the cabin in which the experiment would take place. Then they were introduced to the first task, which was a Slider task (Gill & Prowse, 2011). This task, which is described for completeness here but is not analysed, requires the placement of sliders that range from 0 to 100 right in the middle point of 50 and takes about 4 min. After practising this task, there would be around a minute-long break plus a calibration before proceeding to record the baseline pupil size. After the baseline recording, the participants would move on to perform the subsequent experimental tasks. While the parents were performing these tasks, the child performed the IQ test and a survey, which took approximately 40 min to complete altogether. When both parent and child were done, they switched places and the child completed the experimental tasks, while the parents completed the IQ test and responded to an additional survey.

The experimental set-up (desk, computer, and eye-tracker) was situated in a cabin inside the lab, isolated from outside light and sound. The height of the participant’s chair was adaptable, so that they could comfortably fit into the chin-rest placed in front of the computer and eye-tracker, meant to keep the position of the head fixed during the experiment. Subjects’ eyes were about 60 cm from the screen. The participants were instructed to maintain a stable head position during the whole duration of the calibration and the baseline recording. The researcher sat outside of the cabin, and communicated with the participant through a camera and audio device. The experimental tasks were programmed using OpenSesame (version 3.2.8: Kafkaesque Koffka; Mathôt et al., 2012).

### Measures

*Baseline pupil size:* Pupil size was recorded using a Tobii Pro Spectrum 150 eye-tracker. The light parameters inside of the chamber were 14 lx of illuminance and 40 cd/m^2^ of luminance during the tasks. These measurements were made with a photometer Delta Ohm HD9921 at the position of the participants’ eyes, 60 cm away from the computer screen subtending 33 (H) × 29 (V) degrees of visual angle. When measured at the screen, the illuminance value was 55 lx. The eye-tracker took measurements of both eyes at a frequency of 150 Hz over 15 s. Data were processed following the recommendations of Mathôt et al. (2018): eliminating invalid and unrealistic data points (pupil sizes less than 1.5 mm or bigger than 9 mm) and all the ± 220 ms recordings from the artifacts. From this measurement, the coefficient of variance (CoV) and mean pupil size were calculated to be used in the analyses.

*Fluid intelligence* was measured using the Raven’s Progressive Matrices (RPM) test (Raven et al., 1996). The RPM consists of five sets of matrices, each set increasing in difficulty, with 12 matrices per set. The test was administered to both parents and children using a computerized version programmed in OpenSesame. Participants began with the first matrix and were instructed to complete as many matrices as possible within the given time limit. Due to time constraints in the experiment, the time limit for this test was restricted to 5 min. The total number of resolved matrices, as well as the proportion of correctly answered matrices as a robustness test, were used as dependent measures.

*Sociodemographic variables* regarding education and employment status were provided by the participants to the recruiting company. Gender was directly asked in the surveys that both parent and child completed and age in months was calculated by asking the participants for their birth month. Regarding the ethnicity of the participant, parents were asked if they were born in Spain and asked to describe their country of birth if otherwise. The non-Spanish participants were originally from South and Central America. They were classified as Latino/a if that was the case.

*Other relevant physiological variables:* Both participants were briefly interviewed by the researcher before the beginning of the experiment. In particular, they were asked about the presence of vision problems and, if needed, what kind of correction they had or would use during the experiment (such as contact lenses, glasses, surgery or none). Their hand dominance was also surveyed and introduced in the analyses, following other replication studies (Ruuskanen et al., 2024). Additionally, information about the day of the week and time of the day was recorded to control for their effect on participants’ fatigue, which could affect their baseline pupil size and variability (Lowenstein et al., 1963).

### Data processing and analysis

For the baseline pupil size measures, reliability was estimated using a first/second half split of the recordings from each sample (children and adults). For the fluid intelligence measure, reliability was assessed using an odd/even split. As, due to practical reasons, a time limit needed to be imposed for this task, some participants performed an odd and others an even number of matrices. To ensure the same number of matrices per participant was included in each subset of the data after splitting, the last response was deleted before splitting for those participants who completed an odd number of matrices. For all reliability measures, the Spearman-Brown split-half correction was applied to the correlation between the halves.

To test hypotheses 1A and 1B, two multivariate regression models were fit. In the first model (1A), the average baseline pupil size of each child was used as the dependent variable, and the total number of responses in the fluid intelligence test was used as the main predictor. The covariates included in the regression model were age in months, gender, hand use (left or right-handed), vision correction dummy (vision corrected or not), day of the week, eye colour (dark or light), and ethnicity. To test H1B concerning the variability of the baseline pupil size, a second multivariate regression model was fit, with the same main predictor and covariates but using the CoV of each child as the dependent variable, as performed by Aminihajibashi et al. (2019).

To test hypotheses 2a and 2b, two additional multivariate regression models were used, following the same model structure but using the parental data. These analyses were performed using STATA version 15 SE (Stata Corp, 2017).

### Statistical approach

Given the inconsistency of effect sizes across studies examining the relationship between fluid intelligence and baseline pupil size, properly calibrating a traditional power analysis would have been challenging, which leaves the question of the power of the study open. To tackle this problem, we also conducted additional Bayesian analyses on the data, to allow for quantification of evidence for the null hypothesis (Dienes., 2014). The same variables were used as in the multivariate regression reported above. These analyses were performed in JASP (version 0.16.4; JASP Team, 2023).

## Results

Regarding the dependent variables, for children, the distribution of average pupil size ranged from 2.85 to 5.53 mm (*M*_*children*_ = 3.85, *SD*_*children*_ = 0.54), while the baseline pupil CoV had a range of 2.34–15.59%, (*M*_*children*_ = 6, *SD*_*children*_ = 2.77). In the fluid intelligence measures, children completed from 7 up to 33 matrices (*M*_*children*_ = 24.75, *SD*_*children*_ = 4.38). For the parents, the pupil sizes ranged from 2.14 to 4.87 mm (*M*_*parents*_ = 3.33, *SD*_*parents*_ = 0.52), while the CoV presented a range of 0.49–12.49% (*M*_*parents*_ = 4.02, *SD*_*parents*_ = 1.79). Parents completed from 14 up to 43 matrices (*M*_*parents*_ = 28.62, *SD*_*parents*_ = 4.99) in the RPM test. The Pearson correlation between the average baseline pupil size and the fluid intelligence score did not indicate a significant association for either children or adults (*r*_children_= 0.08, *p* = 0.45, *r*_parents_ = −0.01, *p* = 0.95). A null effect was likewise found for the relationship between fluid intelligence and baseline pupil CoV (*r*_children_= 0.10, *p* = 0.32, *r*_parents_ = −0.03, *p* = 0.77).

**Fig. 1:**
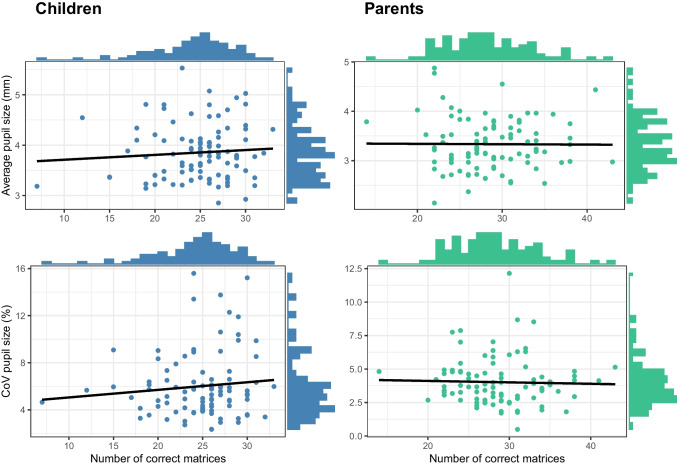
Relationship between fluid intelligence and pupil. Two-way scatterplots of the relationship of fluid intelligence, measured as the number of correct matrices in the RPM test, with average pupil size in mm (top row) and pupil variability measured as the pupil baseline CoV (bottom row). Variable distributions can be found at the margins

For children, the reliability estimates of the dependent variables were 0.95 for the average baseline pupil size and 0.66 for the baseline pupil CoV, while the reliability estimate for the fluid intelligence measure was 0.87. For parents, the reliability estimates of the dependent variables were 0.97 for the average baseline pupil size and 0.55 for the baseline pupil CoV, while the reliability estimate for the fluid intelligence measure was 0.84 Fig. 1.

For the confirmatory analyses, four multiple linear regression models were fit in order to test the correlation between fluid intelligence and both average baseline pupil size and baseline pupil variance (measured as the baseline pupil CoV) for both children and parents, while taking various other factors into account. The results of these analyses are presented in Table 1. According to these analyses, fluid intelligence did not correlate with either the average baseline pupil size or the baseline pupil size variability. Regarding the baseline pupil variability, sex was a significant predictor of pupil variability among children, with female participants presenting 2.24% more variability than male participants on average. For the adults, participants who were born in Latin American countries presented a higher coefficient of variation than Spanish born counterparts on average. The results presented in Table 1 belong to models in which fluid intelligence is measured as the total correct answers in the Raven Progressive Matrices test. For robustness, these models were replicated using the proportion of correct answers from the total matrices answered as a measure of fluid intelligence. Results are in Table S1 in the Appendix (Online Supplemental Material). All the results described in this section and presented in Table 1 are replicated in Table S1 (Online Supplemental Material).

**Table 1:** Multivariate regression models predicting average pupil size

	Average pupil		CoV	
	Children	Parents	Children	Parents
Fluid intelligence	0.000 (0.013)	−0.002 (0.012)	0.028 (0.074)	−0.056 (0.037)
Age	−0.005 (0.009)	−0.002 (0.001)	0.045 (0.054)	−0.005 (0.003)
Sex (ref.: Female)				
Male	0.086 (0.126)	−0.137 (0.142)	−2.235 (0.688)**	−0.005 (0.438)
Hand dominance (ref: Right)				
Left	−0.362 (0.187) †	0.187 (0.276)	1.460 (1.016)	0.868 (0.854)
Vision corrected (ref. No)				
Yes	−0.253 (0.177)	0.017 (0.144)	1.182 (0.963)	−0.333 (0.444)
Week day (ref.: Monday)				
Tuesday	−0.382 (0.180)*	0.016 (0.189)	0.237 (0.979)	0.067 (0.583)
Wednesday	−0.082 (0.197)	0.163 (0.191)	−0.541 (1.03)	0.344 (0.589)
Thursday	−0.529 (0.185)**	−0.151 (0.190)	−0.051 (1.01)	0.258 (0.587)
Friday	−0.143 (0.196)	0.171 (0.207)	0.384 (1.06)	1.149 (0.641) †
Eye color (ref.: Dark)				
Light	0.138 (0.154)	0.026 (0.144)	0.469 (0.837)	−0.523 (0.445)
Time of the day	0.074 (0.083)	−0.015 (0.076)	−0.157 (0.454)	0.079 (0.235)
Ethnic minority status (ref. Majority)				
Latin	0.128 (0.165)	0.313 (0.176) †	−0.225 (0.901)	−1.40 (0.544)*

Standard errors in parentheses. † p < 0.1, * p < 0.05, ** p < 0.01, *** p < 0.001.

Additional Bayesian analyses also indicate a lack of relationship between fluid intelligence and baseline pupil size. For children, the Bayesian correlation analysis provided support for the null hypothesis for both average pupil size (*BF01*_*children*_ = 6.66) and CoV (*BF01*_*children*_ = 5.99). Further, the Bayesian regression with average pupil size as dependent variable, and the same covariates as specified above, supported the null model as the model that fitted the data best. When predicting baseline pupil size variability for children, the best model included handedness and sex, but not fluid intelligence. For parents, Bayesian correlations again did not provide support for the null hypothesis for average pupil size (*BF01*_*parents*_ = 7.41) or variability (*BF01*_*parents*_ = 2.58). And the Bayesian regression analysis supported the null model as the best model for explaining both average pupil size and variability in pupil size.

## Discussion

The present study aimed to contribute to the literature investigating the relationship between fluid intelligence and baseline pupil size and pupil variability. This line of research emerged after empirical evidence was provided for a positive relation between average pupil size at rest and both fluid intelligence and WMC (Tsukahara et al., 2016). While some studies have found evidence of a positive intelligence-pupil size association (Heitz et al., 2008; Van Der Meer et al., 2010), and Aminihajibashi et al. (2019) found an association between pupil size variability and fluid intelligence, most studies later failed to replicate these results, and a recent meta-analysis found a near zero correlation in the relation between WMC and pupil size (Unsworth et al., 2021). However, previous studies have focused on adult populations, and particularly on university students, which are relatively homogenous in terms of their ages and cognitive abilities. Given the theoretical and practical relevance of being able to objectively measure fluid intelligence through pupil measures, it is a crucial question whether this relationship holds in other demographics. Here we describe results from a pupillometry experiment conducted among almost a hundred dyads of parents and their children to fill this gap. Specifically, we tested this relationship in a sample of fifth-grade children around age 10 years, as well as their parents, in a sample that was representative of the general population in terms of socioeconomic status.

As with most previous attempts to replicate, we also did not find any evidence for a relationship, neither among parents nor among children, when examining both average resting-state pupil size and pupil variability. This suggests that the relationship between baseline pupil size and pupil-size variability on the one hand, and fluid intelligence on the other, is either very small or non-existent. However, since the data used in this paper were not collected with the original purpose of testing these specific hypotheses, there are some limitations that need to be addressed.

The first limitation is that the baseline of the study was taken after trial performance on a Slider task (Gill & Prowse, 2011). Although there was around a 1-min break plus a calibration before proceeding to record this baseline, and although the Slider task is exceedingly simple, it could be that there are individual differences in the measured baseline that are a product of differences in arousal as a reaction to having practised the Slider task (and altered expectations) that could be adding noise to the baseline pupil size measure. The second limitation is that data collection was not designed following all aspects of Tsukahara and Engle’s recommendations (2021b). While their concern regarding homogeneity in the sample and ability is resolved by including two different cohorts in the study, as well as by having a representative sample, other recommendations were not followed. The main recommendation that Tsukahara and Engle give in their response to previous replication studies pertains to the lighting conditions; specifically, they recommend a dark experimental setting to ensure that there is sufficient variability in the baseline pupil size measure. Our illuminance setting falls somewhere in between their dim and bright settings: the illuminance measure for the participant perspective falls between the indicated range they call “dim” of 1–15 lx with the lights off, while the illuminance in the screen falls above this range (Tsukahara & Engle, 2021b). However, with a mean pupil size of 3.22 mm and a standard deviation of 0.49 mmm, the distribution of pupil sizes for both children and parents are closer to the ones reported by other replication attempts (e.g., see Unsworth et al., 2021) than the distribution reported in the original study by Tsukahara et al. (2016) or in Tsukahara and Engle (2021a, 2021b).

In conclusion, this study featuring a child sample adds crucial evidence to the current debate about the relationship between fluid intelligence and resting pupil size or pupil-size variability. Specifically, our results do not support the existence of such a relationship, using a representative sample consisting of both adults and children, thus addressing one of the possible reasons for failed replications mentioned by Tsukahara and Engle. The use of a child population and a quota sample that guarantees a balanced population in terms of socioeconomic background is a novel contribution to this debate. However, since our study design of the data collection was not originally meant to test this particular relationship, some of Tsukahara and Engle’s recommendations were not fully met. Hence, future studies that want to perform a replication on children, or other pupillometry studies, could take closer consideration of the recommendations when designing the study to be able to provide additional evidence.

## Supplementary Information

Below is the link to the electronic supplementary material.

Supplementary file1 (DOCX 23 KB)

## Data Availability

Data for the experiment are available upon request.

## Footnotes

1 More details about this study can be found at: https://www.effort-project.eu/.

## Code availability

All the programs and code used for this manuscript are available upon request.
