# Supplementary material for: No evidence for association between pupil size and fluid intelligence among either children or adults
Source: Psychon Bull Rev. 2025 Feb 26;32(4):1795–802. doi: 10.3758/s13423-025-02644-2 (PMC12325416; doi:10.3758/s13423-025-02644-2)
Supplement: Supplementary file 1 — Supplementary file1 (DOCX 23 KB) [file 13423_2025_2644_MOESM1_ESM.docx]

**Appendix**

**Table S1**

| *Multivariate regression models predicting average pupil size with proportion of correct answers* | | | | | | | |
| --- | --- | --- | --- | --- | --- | --- | --- |
|  | Average pupil size | | | CoV | | | |
|  | Children | | Parents | Children | | Parents | |
| Fluid intelligence | .010(.375) | | -.361(.474) | .889(2.04) | | -.802(1.48) | |
| Age | -.005(.009) | | -.001(.001) | .042(.055) | | -.004(.003) | |
| Sex (ref.: Female) | |  | | |  | |  |
| Male | .085(.126) | | -.144(.143) | -2.290(.675)** | | -.134(.437) | |
| Hand dominance (ref: Right) | | | | | | | |
| Left | -.363(.188) † | | .201(.276) | .791(765) | | .864(.868) | |
| Vision corrected (ref. No) | | | | | | | |
| Yes | -.254(.177) | | .013(.143) | 1.137(.960) | | -.393(.449) | |
| Week day (ref.: Monday) | | | | | | | |
| Tuesday | -.383(.180)* | | -.002(.190) | .240(.979) | | .028(.597) | |
| Wednesday | -.081(.195) | | .154(.190) | -.464(1.060) | | .413(.597) | |
| Thursday | -.528(.188)** | | -.175(.191) | -.008(1.024) | | .285(.604) | |
| Friday | -.143(.199) | | .196(.209) | .443(1.080) | | 1.209(.659) | |
| Eye color (ref.: Dark) | | | | | | | |
| Light | .138(.154) | | .022(.144) | .481(.839) | | -.573(.451) | |
| Time of the day | .074(.083) | | .010(.076) | -.157(.454) | | .067(.239) | |
| Ethnic minority status (ref. Majority) | | | | | | | |
| Latin | .128(.165) | | .311(.175) † | -.226(.899) | | -1.33(.551)* | |

*Note.* Robust standard errors in parentheses. †p< .1. *p< .05. **p< .01. ***p< .001.
